# Deep-Learning-Based Real-Time Passive Non-Line-of-Sight Imaging for Room-Scale Scenes

**DOI:** 10.3390/s24196480

**Published:** 2024-10-08

**Authors:** Yuzhe Li, Yuning Zhang

**Affiliations:** 1 School of Electronic Science and Engineering, Southeast University, Nanjing 211189, China; 220231895@seu.edu.cn; 2 Shi-Cheng Laboratory for Information Display and Visualization, Nanjing 210013, China

**Keywords:** NLOS, USEEN, diffuse surfaces, room-scale scenes, privacy-preserving security monitoring system

## Abstract

Non-line-of-sight imaging is a technique for reconstructing scenes behind obstacles. We report a real-time passive non-line-of-sight (NLOS) imaging method for room-scale hidden scenes, which can be applied to smart home security monitoring sensing systems and indoor fast fuzzy navigation and positioning under the premise of protecting privacy. An unseen scene encoding enhancement network (USEEN) for hidden scene reconstruction is proposed, which is a convolutional neural network designed for NLOS imaging. The network is robust to ambient light interference conditions on diffuse reflective surfaces and maintains a fast reconstruction speed of 12.2 milliseconds per estimation. The consistency of the mean square error (MSE) is verified, and the peak signal-to-noise ratio (PSNR) values of 19.21 dB, 15.86 dB, and 13.62 dB are obtained for the training, validation, and test datasets, respectively. The average values of the structural similarity index (SSIM) are 0.83, 0.68, and 0.59, respectively, and are compared and discussed with the corresponding indicators of the other two models. The sensing system built using this method will show application potential in many fields that require accurate and real-time NLOS imaging, especially smart home security systems in room-scale scenes.

## 1. Introduction

Non-line-of-sight (NLOS) imaging technology, which involves reconstructing invisible scenes behind obstacles or around corners, is significant work and can be applied to numerous fields, such as anti-terrorism, remote sensing, autonomous driving, and medical imaging [1]. Despite its potential for widespread use, it remains one of the most challenging topics due to the difficulty of characterizing the diffuse surface thoroughly, which arises from its unknown and random nature [2].

In modern smart home systems, security monitoring is a key link [3]. Traditional surveillance systems rely on cameras to capture images directly within the line of sight, but this may be limited in some cases, such as obstructions in the room or privacy protection requirements. For covert monitoring, NLOS imaging devices can be installed on the walls or ceilings of the room to detect activities in the room using reflections from the walls or furniture. This device can provide covert monitoring capabilities by receiving and analyzing reflected light or signals to identify and track activities. In terms of indoor navigation and positioning, NLOS sensors installed in the room can help the system accurately locate objects or people in the room, even if they are blocked by furniture or other obstacles [4]. This is very useful for applications such as the automation of smart home systems and indoor robot navigation. It can be seen that the research on non-line-of-sight imaging at the room scale is of great significance for security monitoring, covert monitoring, and promoting the development of smart home systems under the premise of protecting privacy.

However, most of the existing non-line-of-sight imaging technologies are based on research in large-scale scenarios. Repasi et al. proposed a long-range target classification system that can penetrate obscurants or vegetation in certain conditions using short-wavelength IR range-gated imaging devices [5]. Velten et al. used time-of-flight (TOF) cameras to capture the indirect light that bounces off a hidden object and then reconstruct its shape using specific algorithms [6]. O’Toole et al. developed a more computationally and memory-efficient NLOS imaging system using confocal scanning [7]. Batarseh et al. utilized the spatial coherence of light scattered from the rough surfaces to build an NLOS imaging system [8]. Generally, these classical approaches can mainly be divided into two categories, one based on TOF and the other based on the coherence of light. These existing non-line-of-sight imaging methods do have advantages in imaging at long distances or in large-scale experimental scenarios, but they have certain limitations and disadvantages when applied to scene reconstruction at the room scale. For one thing, the performance of the TOF-based NLOS imaging systems is highly dependent on the spatiotemporal resolution of the devices [6,9], which means that precise and expensive instruments are required, which greatly increases the power consumption and cost. In addition, although TOF-based systems exhibit the ability to reconstruct room-scale hidden scenes [10], they struggle to achieve real-time reconstruction at a certain rate, which severely limits the applicability of the system for room-scale imaging. For another, the performance of the methods using the coherence of light depends on the strength of the scattering [8,11], which is closely related to the distance from the wall to the target [12]. This may make it challenging to apply these methods to scenes of room-scale size. Currently, there is little research on non-line-of-sight imaging in room-scale scenes.

To address the above challenges, scholars have proposed a variety of methods. Recently, it has been demonstrated that deep learning (DL) is a powerful tool for solving challenging imaging inverse problems [2,13,14,15,16,17,18,19,20], including NLOS imaging. The passive methods based on DL can simplify data acquisition, which is advantageous in many practical applications [21,22] and can quickly reconstruct hidden scenes at room scale. There are several neural networks proposed for NLOS imaging. Li et al. developed a convolutional neural network to generalize and make high-quality object predictions through a different set of diffusers of the same class [23]. Saunders et al. proved that the penumbra cast by an object might contain enough information for reconstruction by realizing 2D color NLOS imaging with an ordinary digital camera [24]. Kim et al. proved that the images of objects could be predicted based on the pre-trained network even when the input data are beyond the category of the training dataset [25]. Wu et al. reported an untrained neural network (UNN), which improved the image quality of NLOS systems [26]. However, these studies commonly utilize display screens or static scenes rather than room-scale hidden scenes with moving objects to conduct experiments. This could be due to the insufficient generalization and feature extraction capabilities of the network. Real-time reconstruction for humans as objects in hidden scenes via DL, which requires high reconstruction speed, high NLOS imaging quality, and suitability for room-scale scenes, has been rarely studied. This relies on the network’s feature extraction and generalization capabilities. Furthermore, specialized optimization for networks is essential when applied to reconstruct hidden scenes to achieve reconstruction results of high quality [22].

In this paper, we propose a deep learning-based passive non-line-of-sight imaging framework to build a sensing system capable of reconstructing room-scale scenes in real time. A convolutional neural network (CNN) is designed and applied for reconstruction. Through experiments, it is verified that this architecture can successfully reconstruct room-scale hidden scenes with persons as objects via diffuse surfaces, even if the objects are not included in the training dataset. Specifically designed and optimized for NLOS imaging, the network exhibits high feature extraction capabilities and generative capabilities and is robust to ambient light interference conditions. Maintaining a limited network scale, the method can reconstruct hidden scenes at a high speed, precisely 12.2 milliseconds per estimation.

## 2. Diffuse Reflection Loss Analysis

**Figure 1:**
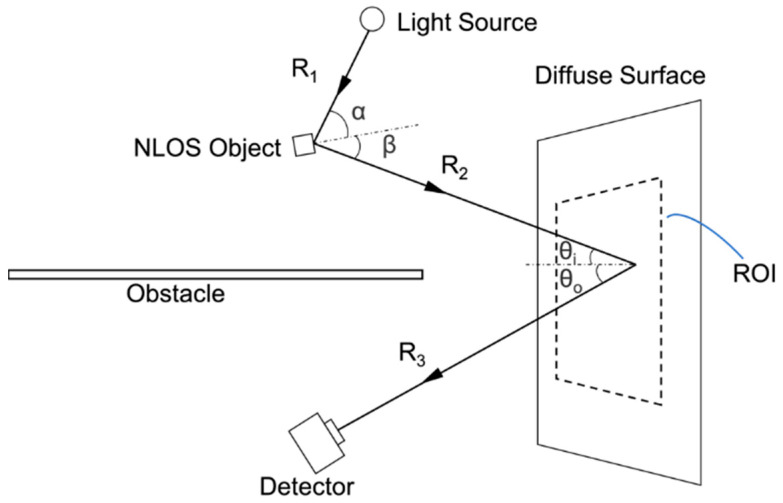
A simplified model for passive NLOS imaging.

We assume that the hidden object is illuminated by a light source in the hidden scene and that the diffuse surface is only illuminated by the light reflected from the hidden object. Figure 1 shows a simplified model for passive NLOS imaging. Based on the assumption that the object and reflection surface are ideal Lambertian reflectors, the luminous flux received by the detector can be calculated as follows:

(1) $$\Phi_{recv}=\frac{\rho_{obj}\rho_{surf}I_{0}A_{\mathrm{obj}}A_{\mathrm{recv}}\cos\alpha \cos\beta }{\pi^{2}{R_{1}}^{2}}\iint_{ROI}\frac{\cos\theta_{i}\cos\theta_{o}}{{R_{2}}^{2}{R_{3}}^{2}}dxdy$$

where $\rho_{obj}$ and $\rho_{surf}$ are the reflection coefficients of the hidden object and the diffuse surface, respectively. $I_{0}$ is the intensity of the light source in the direction of illuminating the hidden object. $A_{obj}$ and $A_{recv}$ are the effective areas of the hidden object and of the detector. x and y are, respectively, the horizontal and vertical spatial coordinates within the range of interest (ROI) on the diffuse surface. α and $\theta_{i}$ are the incident angles of the diffuse reflection on the hidden object and on the diffuse surface. β and $\theta_{o}$ are the exit angles of the diffuse reflection on the hidden object and on the diffuse surface. $R_{1}$, $R_{2}$, and $R_{3}$ are, respectively, the distance between the light source and the hidden object, the distance between the hidden object and the reflecting point on the diffuse surface, and the distance between the reflecting point on the diffuse surface and the detector.

As can be seen from Equation (1), the luminous flux received by the detector is negatively related to $R_{1}$, $R_{2}$, and $R_{3}$, that is, the scale of the hidden scene. Also, it is positively related to the luminance of the hidden objects, which is decided by the intensity of the light source and the distance between the hidden object and the light source if the hidden object is not self-luminous. Generally, it is difficult for the hidden objects illuminated by light sources used for indoor illumination to have the same luminance as LCD screens. Therefore, compared to the studies using smaller-scale scenes or scenes displayed by LCD screens, a passive room-scale NLOS reconstruction requires a system with stronger feature extraction capabilities and generative capabilities.

## 3. Method

### 3.1. Experimental Setup

**Figure 2:**
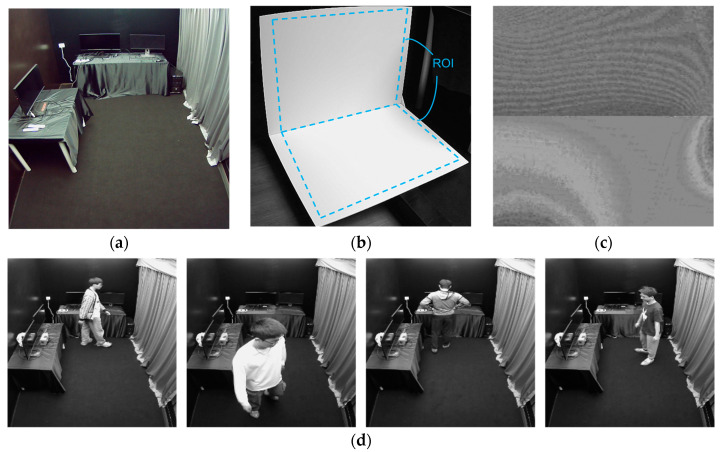
(**a**) The room utilized as the hidden scene; (**b**) A diffuse reflection image before the preprocessing procedure. The framed area represents the ROI; (**c**) A diffuse reflection image after the preprocessing procedure; (**d**) Partial label images from the training dataset and validation dataset.

A room with dimensions of 4.5 m in length and 2.4 m in width and height, illuminated by six light-emitting diode (LED) lamps on the ceiling, each with a power of 60 W, was utilized as the hidden scene; see Figure 2a. The scene was laid out in black. Thus, two polyvinyl chloride (PVC) expansion sheets were used as diffuse reflection surfaces; see Figure 2b. Two consumer-grade CMOS cameras from Rayvision were used to capture datasets. The viewing angle of both cameras is about 70°. One located outside the scene was responsible for photographing the diffuse surface. The other one was placed at the top inside the hidden scene. A total of 11 volunteers took part in the dataset collection, with data from 3 participants used as the testing set and the remaining data used as the training set. The data from one of the participants whose data are used as a part of the testing set is slightly different because an extra external light illuminates the diffuse surface as interference during the capturing process. The robustness to ambient light interference conditions on the diffuse surface of the proposed method will be analyzed in Section 5 based on the data from this participant. Each participant was asked to walk and pose randomly within the hidden scene, and 5000 sets of photos were captured for each one of them. Before training, 10% of the dataset was randomly selected and excluded from the training set as the validation dataset. Therefore, the training dataset, validation dataset, and test dataset contain 36,000 sets, 4000 sets, and 15,000 sets of images, respectively. Figure 2d shows partial label images from the training and validation datasets.

Before being inputted into the neural network, to minimize the unwanted disturbances caused by ambient light and improve the data quality [19], all of the diffuse reflection images have gone through a preprocessing procedure based on OpenCV, a computer vision library. The detailed process is as follows: Firstly, the grayscale diffuse reflection images of the hidden objects were subtracted by the grayscale diffuse reflection image without objects. This is to eliminate the background noise on the CMOS and also to better extract differences between diffuse reflection patterns resulting from different hidden objects and their positions [2]. Secondly, the ROI containing effective diffuse information was extracted and transformed from an oblique perspective into a positive perspective by the perspective transformation function in OpenCV. During this process, the space bandwidth of each diffuse reflection image was changed to 256 pixels by 256 pixels. Thirdly, and lastly, the images went through a sigmoid function, which can map the input ranging from −1 to 1 to the output ranging from 0 to 1 for image enhancement [19,27]. After undergoing the preprocessing procedures illustrated above, the image can be eventually sent into the neural network. Figure 2c shows a diffuse reflection image after the preprocessing procedure.

### 3.2. Network Architecture

We propose a specific unseen scene encoding enhancement network (USEEN) for hidden scene reconstruction, which combines the U-Net network with the generative adversarial network (GAN). The global consistency advantage of GAN can make up for the shortcomings of U-Net in global visual quality, while the local detail processing ability of U-Net can improve the performance of GAN in regard to details. The final generated image can achieve a good balance between details and global structure, thereby improving the quality of image generation and processing tasks. The GAN is based on game theory, which means that the competition between 2 networks (the generator and the discriminator, respectively) can help significantly improve the quality of the generated images [28]. Unconditioned models cannot control the modes of the generated images, which contradicts the one-to-one correspondence between the images of the diffuse reflection and the hidden objects. A conditional generative adversarial network (CGAN) was applied, which allows the generator and the discriminator to be conditioned on some extra information [29,30], in our case, the preprocessed diffuse reflection images. Thus, the introduction of the discriminator can not only improve the NLOS imaging quality but also improve the accuracy of the output by determining whether the diffuse reflection image matches the reconstructed hidden scene.

**Figure 3:**
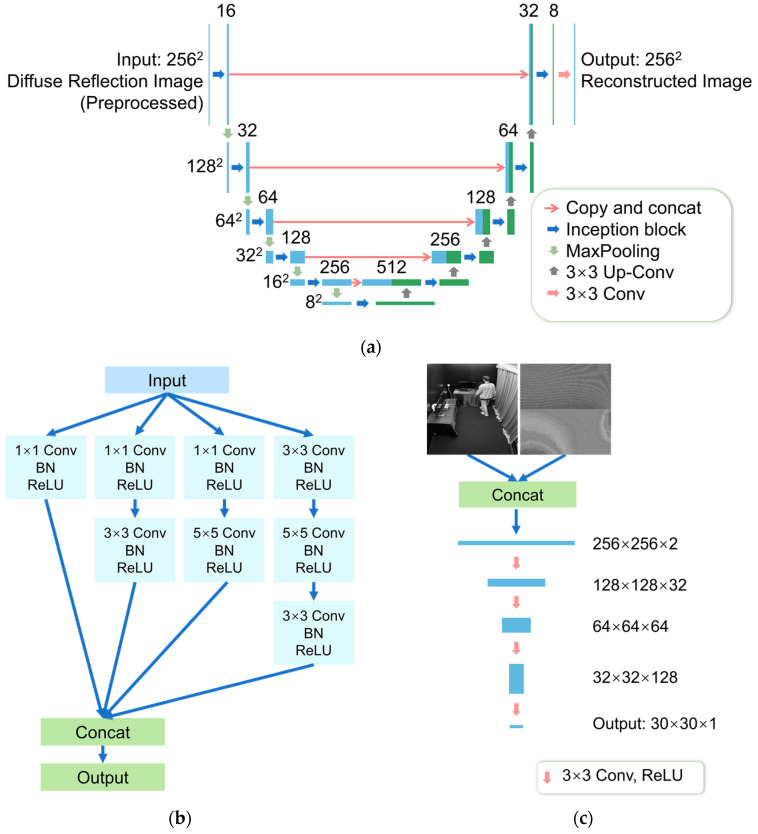
(**a**) The structure of the generator. Each blue box corresponds to a multi-channel feature map and the number of channels is denoted on top of the box; (**b**) The structure of an inception block; (**c**) The structure of the discriminator.

The structure of the generator, which is an improved U-Net, is shown in Figure 3a. Compared with the original U-Net [31], the number of channels of each feature map is reduced, and the convolutional layers in the contracting path are replaced by inception blocks as a means to enhance its feature extraction ability. Its structure is shown in Figure 3b. The core idea of inception block is to capture different scales of input images in the same layer through parallel convolution operations (using different convolution kernel sizes, such as 1 × 1, 3 × 3, 5 × 5), regularization operations, and nonlinear activation operations. This allows the network to extract multiple different features at each layer and fuse them. The introduced 1 × 1 convolution can reduce the amount of computation while maintaining important features. Each inception block concatenates outputs from 4 convolutional paths [32,33]. This structure includes convolutional layers of four different kernel sizes to capture multi-scale features from the diffuse reflection image, thus showing significantly high feature extraction capabilities. The addition of 1 × 1 convolutional layers allows for not just increasing the depth but also the width of our networks without a significant performance penalty [34]. It can capture more dimensions of information and make the model more capable of processing complex scenes. It has been proved that adding a 1 × 1 convolutional layer before convolutional layers with larger kernel sizes helps to propagate information through the network [33]. It has also been proved that the inception blocks are especially useful in the contexts of localization and object detection [32,35], which are both of significance to room-scale NLOS imaging. The application of inception blocks also helps reduce the network size and improve the reconstruction speed while ensuring high imaging performance.

The discriminator, a patch-based fully convolutional network, outputs a 30 × 30 matrix to evaluate each patch of the concatenation of the diffuse reflection image and the hidden scene image. When the NLOS object image is considered to be the corresponding hidden scene image, the output is supposed to be an all-one matrix. Otherwise, the output is expected to be an all-zero matrix. This discriminator, whose structure is shown in Figure 3c, can ensure high-frequency correctness of image reconstruction. As a component of a CGAN, the input of the discriminator consists of both the diffuse reflection images and the hidden scene images, and the discriminator can ensure the correspondence between them.

The objective of the discriminator is to distinguish whether the hidden scene image is generated or real-shot and whether it corresponds to the diffuse reflection image. Assume that x is the diffuse reflection image sent into the generator, y is the ground truth of the hidden scene, and G(x) is the output of the generator, which is the reconstructed hidden scene images. The loss function for the discriminator is as follows:

(2) $$L_{D}=E_{x,y}\left[\log D\left(x,y\right)+\log\left(1-D\left(x,G(x)\right)\right)\right]$$

Among them, D(x,y) represents the probability that the discriminator thinks that the input diffuse image is a real image, and D(x,G(x)) represents the probability that the discriminator thinks that the input diffuse image is the output image of the generator. From the perspective of the discriminator, the discriminator hopes to be able to completely correctly identify all real data and generated data, that is, under ideal conditions, D(x,y) tends to 1 and D(x,G(x)) tends to 0, that is, the discriminator loss $L_{D}$ tends to 0.

The loss function for the generator consists of adversarial loss, which means the generator is trained to fool the discriminator by maximizing $L_{D}$ and reconstruction loss, which is an L1 loss between the reconstructed image and the label image. It was proved that the combination of adversarial loss and reconstruction loss is beneficial because the reconstruction loss itself may prefer a blurry output rather than accurate textures, while the addition of adversarial loss can solve this problem by increasing high-frequency correctness. Meanwhile, the reconstruction loss can ensure low-frequency correctness [29,36]. The loss function for the generator combining adversarial loss and reconstruction loss is as follows:

(3) $$L_{G}=E_{x,y}\left[\log\left(1-D\left(x,G\left(x\right)\right)\right){+\lambda \left\|y-G\left(x\right)\right\|}_{1}\right]$$

From the perspective of the generator, the generator hopes to generate data that are as realistic as possible, so that the discriminator cannot distinguish whether the data are real or generated. In other words, ideally, D(x,G(x)) tends to 1 and the generator loss $L_{G}$ tends to negative infinity, which means that the generator generates perfect fake data. Among them, y − G(x) reflects the difference between the generator’s output and the true value. By introducing the weight λ, the low-frequency information of the generator loss can be adjusted. In our case, the initial value of λ is 10. Through experimental testing, the weight is reduced by 0.8 each time during training and gradually reduced to 0.4 during training, at which time the model training effect is best. This is because the capabilities of both the generator and discriminator are insufficient to make the adversarial loss effective during the initial stages of training. L1 loss can constrain the reconstructed images of the generator to quickly approach the label images, that is, the model can converge quickly in the low-frequency stage. As training progresses, the performance of the generator will gradually improve. At this point, gradually reducing the weight of L1 loss can help the generator focus more on the adversarial loss, that is, pay more attention to high-frequency information. During the training process, the Adam optimizer is used, with the learning rate set to 0.0002 for the generator and 0.0001 for the discriminator.

## 4. Results

**Figure 4:**
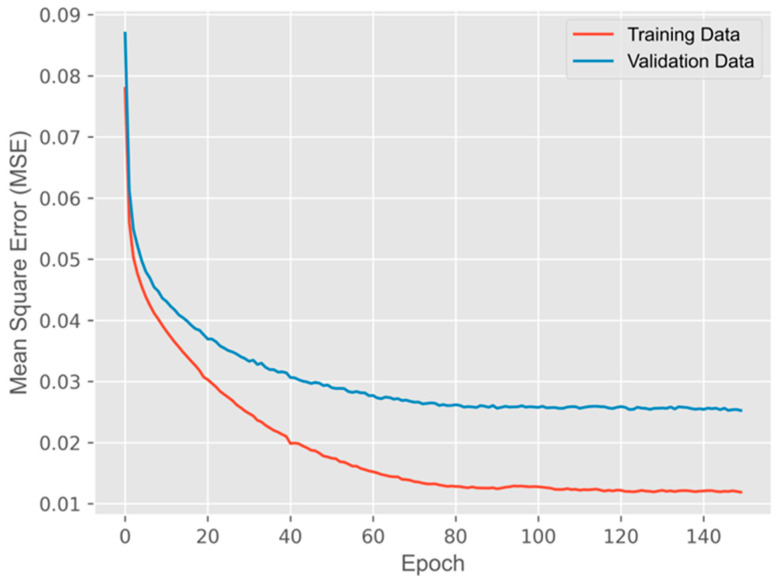
Learning curve of the USEEN model showing the mean square error (MSE) between the reconstructed images and real-shot hidden scenes as a function of epochs.

Our experiment consists of three phases: training, validation, and testing. The acquisition method of each dataset is described in Section 3. The space bandwidth of each input image and label image is 256 pixels × 256 pixels. It should be noted that each label image was downsampled from an original image whose resolution is 640 pixels × 480 pixels. Before the training process, the training dataset was randomly shuffled after concentration. During each epoch, the model outputted the predicted images of the validation dataset after a round of training. After the whole training and validation process, the pre-trained model was evaluated using the test dataset. The whole process was finished using an Nvidia GTX 1080 Ti. The manufacturer is Nvidia Corporation, located in Santa Clara, California, United States. The experimental platform was equipped with Anaconda and TensorFlow. The USEEN was trained for a total of 150 epochs. The average training time for each epoch was approximately 96 s. The mean square error (MSE) between the reconstructed images and real-shot hidden scenes for the training data and validation data was recorded during the training process, see Figure 4. As can be observed, the validation MSE is in good agreement, indicating a well-fit model. The peak signal-to-noise ratio (PSNR) values for the training, validation, and test dataset are, respectively, 19.21 dB, 15.86 dB, and 13.62 dB, and the average values of the structural similarity index (SSIM) are 0.83, 0.68, and 0.59, respectively, which can satisfy the requirement of generating images that are close to the real images.

**Figure 5:**
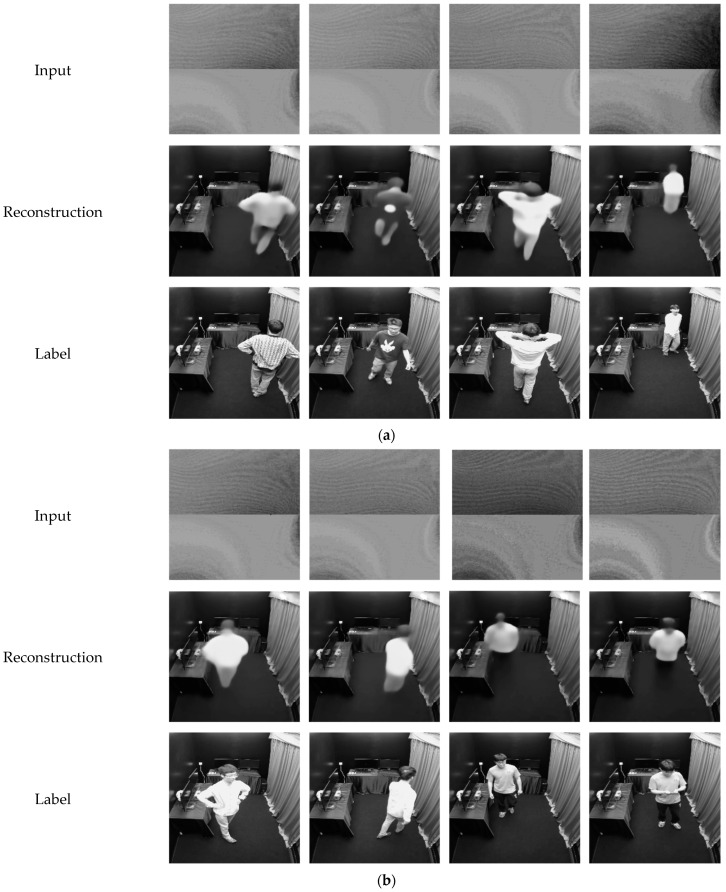

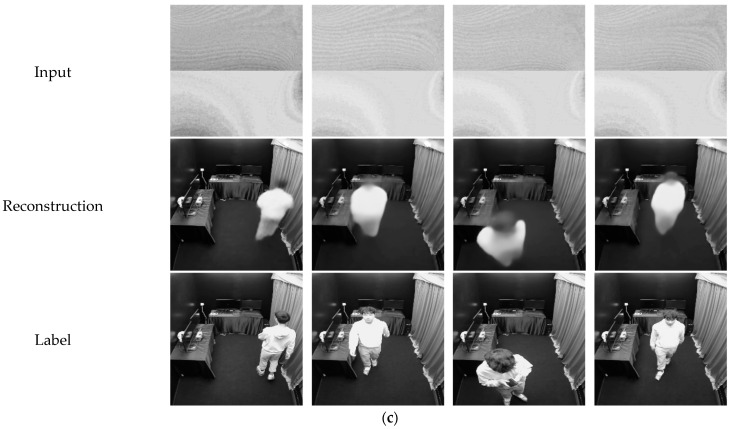
Reconstruction result from the validation and test dataset. (**a**) Partial reconstruction results of the validation dataset; (**b**) Partial reconstruction results of the test dataset; (**c**) Robustness to light interference conditions on the diffuse surface.

The reconstruction results of the partial validation dataset are shown in Figure 5a. As can be seen, due to the fact that the data of the objects has been trained, the reconstruction results of the validation dataset show high performance. The positions and limb poses of the hidden objects could be reconstructed and the reconstructed images include detailed texture. In comparison, the reconstruction results of the test dataset could not include detailed texture information of the hidden objects due to the reason that none of the data from these objects have been trained before. Nevertheless, the position and the clothing grayscale could be reconstructed correctly. The above-average SSIM result data just verify this point. See Figure 5b. This shows the feature extraction ability and generalization ability of the USEEN. It should be noted that the reconstruction time of the proposed USEEN is 12.2 milliseconds per estimation, which can be applied for real-time imaging.

To evaluate the robustness of the USEEN, during the test dataset collection stage of a certain participant, the diffuse surface was illuminated by an additional LED lamp which was placed out of the hidden scene. This lowers the signal-to-noise ratio (SNR) of the input images. As can be seen from Figure 5c, because of the decrease in the SNR, the input images become brighter and the stripes in the images become less prominent compared with input images in Figure 5a,b, thus requiring a higher generalization ability of the imaging system. It can be seen that though the experimental conditions change a bit, the positions, poses, and clothing grayscale of the hidden object can still be reconstructed without additional training. This shows that due to its generalization ability, the proposed USEEN shows robustness to ambient light interference conditions on the diffuse surface.

**Figure 6:**
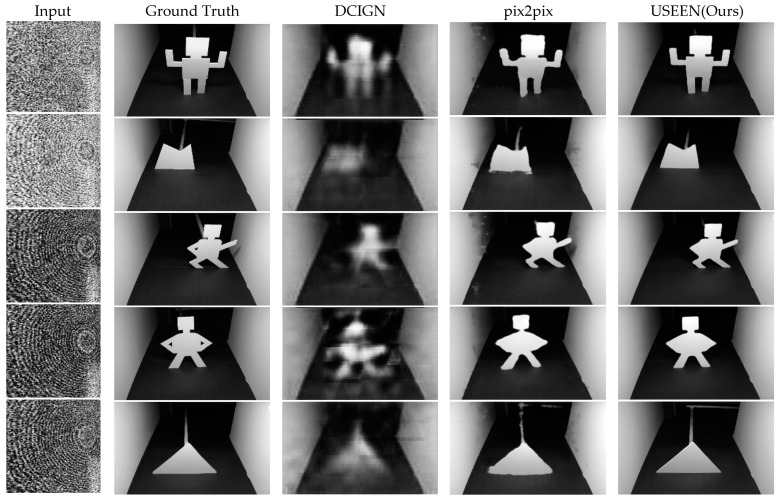
Reconstruction performance comparison among the DCIGN, the pix2pix, and the USEEN.

The results of the reconstruction capability comparison of USEEN with two other image reconstruction networks, the deep convolutional inverse graphics network (DCIGN) and the pix2pix model, are shown in Figure 6. It should be noted that not all test images are included in the training dataset, and all networks have undergone sufficient training iterations to achieve the best performance.

To further evaluate the performance of these networks, we use peak signal-to-noise ratio (PSNR) and structural similarity index (SSIM) to evaluate the difference between the reconstructed images and the real NLOS images; see Table 1.

## 5. Discussion

In order to further verify the robustness of the USEEN network to ambient light interference, we introduced additional external LED light sources in the experiment. The above experimental results show the robustness of the USEEN network to ambient light interference, which further proves the potential of our method in practical applications. In addition, under the conditions of slowly changing natural light, low light, or mixed-light interference, the above experimental conclusions show that the system obviously still has good robustness, but the system may not have good anti-interference ability under high-frequency noise (such as instantaneous strong light interference). This is because instantaneous light interference is irregular and occurs in a short time. If the training data do not contain enough scenes related to light interference, it is difficult for the model to learn and adapt to these interferences from the existing data, and it is difficult to make accurate predictions when facing instantaneous light interference. At the same time, more complex noise interference may require specific physical modeling or optical processing in the imaging system and cannot only rely on data-driven deep learning models. In the future, this study will add scenes containing various light interferences during the training process to improve the model’s adaptability to these noises and at the same time add optical filters or light interference suppression mechanisms to the imaging system to reduce the impact of interference on the signal.

In this study, we compared the reconstruction capabilities of USEEN with two other image reconstruction networks, deep convolutional inverse graphics network (DCIGN) and pix2pix network model. DCIGN is a deep learning model that aims to infer the 3D structure and underlying physical properties of scenes from images and learn interpretable representations of images. The DCIGN model consists of multiple layers of convolution and deconvolution operators and is trained using the stochastic gradient variational Bayes (SGVB) algorithm [37]. Our previous work attempted to use this network to reconstruct images of occluded targets [38]. DCIGN can output reconstructed images and position information with sub-centimeter accuracy at a very high speed, precisely 3.0 milliseconds per estimation. It exhibits high performance, though there is still some room for optimization. For one, the reconstructed NLOS images using the network remained vague. For another, the NLOS objects used in the experiments were 2D models, and the NLOS scene was confined to a cardboard-enclosed area. Therefore, though providing a real-time method for NLOS imaging, this model can hardly meet the requirements of practical applications in some cases. The image translation network pix2pix is an image-to-image translation model based on generative adversarial networks that is very effective at synthesizing photos from label maps, reconstructing objects from edge maps, and colorizing images, among other tasks, but requires paired training datasets and large computational resources. Therefore, in our work, we designed corresponding experiments based on the above network, using an ordinary COMS camera to simultaneously achieve target imaging and dynamic tracking, and compared the reconstruction capabilities of the USEEN network in this study. From the above results, it can be seen that all these three networks can reconstruct NLOS images. Limited by the network size, the generative ability of the DCIGN is narrow. Thus, the reconstructed images by the DCIGN are vague. The poses or shapes of the NLOS objects can roughly be seen. By contrast, the pix2pix network demonstrates impressive reconstructive ability. The images reconstructed by the pix2pix show sharp edges, though they are also a bit distorted, with some irregular noise in the background. The reconstructed results by the USEEN are the clearest and the most detailed among these three networks. The shapes and the positions of the reconstructed NLOS objects are closer to the real-shot NLOS object images. The results of the three evaluation indicators in Table 1 also verify the results we have seen in Figure 6. The image quality of the USEEN is the best among these three networks, while the reconstruction speed maintains high, precisely 12.2 milliseconds for each NLOS reconstruction. This shows that, specially optimized for NLOS imaging, the feature extraction capabilities and generative capabilities of the USEEN are improved when applied to NLOS reconstruction compared with our previous work and a common image translation network, pix2pix. This explains why the USEEN can be applied to a more challenging room-scale scene.

The above experimental results show that the non-line-of-sight imaging system of this study has excellent image reconstruction capabilities on simple diffuse reflection surfaces, but may be affected by multiple scattering, signal attenuation, noise increase and other factors on complex diffuse reflection surfaces. The irregular reflection and multiple scattering effects of complex surfaces may make it difficult for the system to accurately reconstruct the image, resulting in blurring, distortion and reduced resolution. This study will subsequently use multipath signal separation technology to reduce the impact of noise and distortion caused by multiple scattering effects on system image reconstruction. In addition, increasing the performance of the hardware can also effectively improve this problem.

In terms of the network hardware platform construction of the non-line-of-sight imaging sensor system, our current research uses a platform built by Nvidia GTX 1080 Ti. The non-line-of-sight imaging monitoring sensor system built with it has the advantages of fast computing speed and high imaging accuracy, but it has disadvantages such as inconvenience and high power consumption. Therefore, in the future work of this study, we will use FPGA boards with arm architecture and large-capacity storage devices such as DDR SDRAM to write the hardware description language of the network forward propagation calculation; complete the hardware framework design and register-level language code design of modules, including the depth camera control module, user command, and data stream transmission buffer module, the forward propagation calculation module of the USEEN network of this study, and the display output; use the existing hardware platform of this study to complete the back-propagation training process; and rewrite the generated training parameters in real time in the FPGA to achieve the effect of fast-forward computing imaging in real time and according to local conditions. The USEEN network of this study will be completely transplanted to the FPGA platform to increase the portability and versatility of the entire non-line-of-sight imaging monitoring sensor system and reduce power consumption. Therefore, deploying the USEEN model of this study to build a system using FPGA can also extend many application-oriented functions. For example, on the one hand, in the smart home ecosystem, the image reconstructed using the platform constructed by the network in this study retains necessary information and does not contain sensitive detailed texture information, which can make security monitoring effective in protecting user privacy. The platform of this study can be used after preliminary training, and only a single security monitoring can be retained to achieve the function of protecting family security, which effectively reduces the cost of use. On the other hand, in the indoor navigation and positioning system, the indoor navigation robot can accept the image reconstructed by the system in this study for a fuzzy fast positioning, thus improving work efficiency. In short, the network in this study can build the monitoring sensor system required in different fields. When monitoring is required but privacy protection is desired, NLOS technology can avoid directly taking personal images and provide the required information by detecting the patterns and movement trajectories of human activities rather than specific images. This study has great application potential in security monitoring sensors and security systems in various fields.

## 6. Conclusions

In conclusion, we realize real-time reconstruction for humans as hidden objects in room-scale hidden scenes using a USEEN model. The pre-trained model shows considerable feature extraction and generalization capabilities and thus is applicable to objects that are not included in the training dataset and is robust to ambient light interference conditions. When applied to a smaller-scale hidden scene with moving objects, the USEEN can improve the NLOS imaging quality even compared with a larger-scale image translation network. In situations where the important scenes cannot be detected directly, our method may provide an opportunity to reconstruct these scenes. Maintaining a limited network scale, the USEEN can reconstruct hidden scenes at a high speed, precisely 12.2 milliseconds per estimation. This shows that the USEEN in this study can efficiently complete the image reconstruction task in a shorter time while ensuring sufficient signal-to-noise ratio and other indicators. This is of great significance for the research of a series of situations that require the real-time reconstruction of room-scale scenes, such as smart home security monitoring sensor systems, indoor navigation and positioning, and rapid emergency response to indoor safety and health hazards while protecting user privacy.

## Figures and Tables

**Table 1:** Comparison of the performance of different networks to reconstruct NLOS images.

Network	PSNR (dB)	SSIM	Reconstruction Time (ms)
USEEN (Ours)	19.209	0.829	12.2
DCIGN	15.452	0.536	3.0
Pix2pix	16.926	0.803	30.9

## Data Availability

Data are self-contained within this article.
